# Effects of a Community-Based Pilot Intervention on Home Food Availability among U.S. Households

**DOI:** 10.3390/ijerph17228327

**Published:** 2020-11-11

**Authors:** Rachel A. Cassinat, Meg Bruening, Noe C. Crespo, Mónica Gutiérrez, Adrian Chavez, Frank Ray, Sonia Vega-López

**Affiliations:** 1 College of Health Solutions, Arizona State University, 550 North 3rd Street, Phoenix, AZ 85004, USA; rachel.cassinat@gmail.com (R.A.C.); meg.bruening@asu.edu (M.B.); moni.gutierrez@asu.edu (M.G.); drachavez31@gmail.com (A.C.); 2 School of Public Health, San Diego State University, 9245 Sky Park Ct. Suite 224, San Diego, CA 92123, USA; ncrespo@sdsu.edu; 3 City of Phoenix, Parks and Recreation Department, 212 E. Alta Vista Rd., Phoenix, AZ 85402, USA; frank.ray@phoenix.gov; 4 Southwest Interdisciplinary Research Center, Arizona State University, 201 N. Central Ave, Room 3346, Phoenix, AZ 85004, USA

**Keywords:** community-based intervention, diet, home food availability, home food environment, sugar sweetened beverages, fruit and vegetable intake

## Abstract

The purpose of this study was to assess the effects of a pilot community-based behavioral intervention on the home food environment in U.S. households. Parents (21 females, 2 males; age = 36 ± 5.5 years; 78% Hispanic) of elementary school-aged children attended a 10-week dietary improvement behavioral intervention targeting an increase in fruit and vegetable consumption and a reduction in sugar intake. Home food availability of fruit, vegetables, and sugar-laden foods and beverages were assessed before and after the intervention using a modified version of the Home Food Inventory. Relative to baseline, the intervention resulted in significant increases in fruit availability (7.7 ± 3.2 items vs. 9.4 ± 3.1 items; *p* = 0.004) and low sugar cereal (2.3 ± 1.4 types vs. 2.7 ± 1.4 types; *p* = 0.033). There was a significant reduction in sugar-sweetened beverage availability (3.2 ± 1.9 types vs. 1.7 ± 1.3 types; *p* = 0.004). There was a significant increase in the number of households with accessible ready-to-eat vegetables and fruit, and a significant reduction in available prepared desserts, and candy (*p* < 0.01). There were no significant changes in the availability of vegetables and sugar-laden cereals. The current intervention resulted in positive changes in the home food environment. Further research to confirm these results in a randomized controlled trial is warranted.

## 1. Introduction

Poor dietary quality, roughly described as low adherence to dietary recommendations [1], plays a role in the development of obesity and chronic diseases [2,3]. While fresh fruit and vegetables aid in the prevention of cardiometabolic diseases [2], sugar intake can trigger weight gain and may play a catalytic role in obesity and chronic disease development [3]. Individuals from ethnically-diverse communities, susceptible to disparities in chronic diseases, often have limited access to fresh fruit and vegetables, and have a greater consumption of sugar-laden foods [3,4].

Hispanics are the largest and fastest growing ethnic minority group in the U.S. [5,6]. Hispanics are a population of concern because of their greater prevalence of chronic disease risk factors such as abdominal obesity, dyslipidemias, and insulin resistance, relative to non-Hispanic Whites and Blacks [7,8,9,10,11,12,13,14]. It has also been reported that Hispanics have a lower overall dietary quality than non-Hispanic Whites [15], further contributing to chronic disease risk. Poor dietary quality is of particular concern among Hispanic youth, as it has been documented that fewer than 10% of Mexican American youths adhere to dietary recommendations regarding fruit and vegetables, fish, whole grains, or sodium intake, and fewer than 35% adhere to sugar-sweetened beverage recommendations [16]. When considering these five components combined, 79–85% of Mexican Americans 5 to 19 years of age had a poor diet score [16]. Given the importance of nutrition for chronic disease prevention, more research is needed to promote healthier dietary intake among this vulnerable population. 

Evidence suggests that interventions which target parents and focus on behavioral change from a family context can improve the parents’ behavior while also improving the behavior of the children in the family, particularly prior to adolescence when parents are still the primary influence on dietary behavior [17,18,19]. This approach has been shown to have positive effects on parent cardiovascular risk factors, parent weight, child weight, and parent- and child-reported eating behaviors [20,21,22,23,24,25]. Conceptually, this approach relies on helping the parents to develop skills related to eating and parenting while encouraging them to model these behaviors, promote healthy behavioral choices in their children and reinforce them, and make changes in the home food environment (HFE) [26]. This intervention approach has the potential to simultaneously affect change at the personal (individual), interpersonal (family) and environmental (home) levels. Currently, there is a lack of evidence to support the use of this model in underserved populations for whom need for behavior change may be greatest [20].

The HFE is a multifactorial concept that comprises the dietary choices available and accessible within a household, as well as the choices around mealtimes that may influence food intake by family members (e.g., media exposure, how meals are served) [27]. The HFE has been suggested as the most influential environmental factor on a child’s dietary intake [28]. Particularly related to dietary choices, two relevant HFE dimensions are home food availability (i.e., the presence or absence of specific foods in the home) and food accessibility (i.e., whether foods are visible and readily obtainable, and therefore more likely to be consumed) [29]. Although it has been suggested that the HFE has a localized and direct impact on food choice and diet quality [30,31,32], the focus of dietary interventions on environmental influences on dietary quality at the family or household level has been limited [26,33]. Behavioral interventions that target the HFE have resulted in significant decreases in energy-dense foods and beverages, and an increase in the amount of fruit and vegetables in the home [34,35]. However, those focusing on ethnically-diverse populations are scarce, and have mainly focused on African-Americans [35]. Therefore, there is a need to assess whether interventions aimed at influencing the HFE are also beneficial in primarily Hispanic populations.

Several HFE measurement tools [29,36,37,38], including a version adapted for low-income Spanish and Somali-speaking households [39], have been validated. However, existing literature involving assessment of the HFE has mostly relied on self-reported data. Studies in which HFE assessment takes place using open inventories (i.e., home visits where home food information is recorded by research staff rather than relying on self-reported data), are scarce [40]. Moreover, open inventories have not been used to assess the efficacy of a behavior change intervention. Therefore, this quasi-experimental pilot study used open inventories to assess the effects of a community-based dietary behavior change intervention delivered to parents of school-aged children on their HFE. Such an assessment is important as it allows for an objective evaluation of whether a behavioral intervention can influence household-level factors that directly influence dietary intake.

## 2. Materials and Methods 

### 2.1. Participants

One adult parent (≥18 years) and one 6–11-year-old child from the same household were enrolled in a 10-week quasi-experimental dietary behavior change pilot intervention delivered concurrently at an inner-city elementary school and a community center in Phoenix, AZ. The study team did not have a say as to which parent within a household enrolled in the study. Parent participation depended on willingness and availability to take part of the intervention sessions. Enrolled parents participated in the behavioral intervention described below, while their children took part in concurrent physical activity sessions that did not include information about the HFE. For the purposes of the current analysis, we only included data from the parent participants given that they were the ones consenting to the home data collection procedures to assess the HFE (see below).

Participants were recruited through the use of flyers distributed at both locations, referrals from prior participants, and word of mouth. Exclusion criteria (for both, parents or children) were: (a) medical conditions requiring a specialized diet (e.g., food allergy, phenylketonuria); (b) participation in a separate diet modification program; (c) fruit and vegetable intake ≥5 servings/day; (d) inability to attend sessions; and (e) pregnancy. The questions used in the screening process to verify participant eligibility are available in Supplementary Material 1. Although the original intent was to exclusively enroll Hispanic families, all interested families were admitted to the study regardless of ethnicity, per request of our community partners where the intervention was delivered. Nevertheless, both locations were located in an area with a large Hispanic population (63%) [41], and most enrolled participants were Hispanic (see results). Sessions at the community center were conducted in Spanish to accommodate the high percentage of Hispanic participants; sessions at the school were delivered in English. For the current report, parent participants had to consent to a home visit for assessment of the HFE. Out of 34 participating families, 27 consented for baseline home visits (19 attending the program at the community center and 8 attending at the elementary school), and 23 completed post-intervention follow-up visits and were included in the present analysis. All participants provided written informed consent both for program participation and the home visits. This study was approved by the Institutional Review Board at Arizona State University (IRB#STUDY00000427 and IRB # STUDY00000267).

### 2.2. Intervention

The 10-week group-based program was delivered once weekly at the inner-city school (90 min/session) and twice weekly at the community center (45 min/session) and included a variety of behavioral modification techniques demonstrated to promote dietary behavior change, particularly targeting an increase in fruit and vegetable consumption and a reduction in sugar intake. A variety of behavioral modification techniques grounded in principles from social-cognitive theory [42] and operant conditioning [43] were used, with particular emphasis on goal-setting, generating social support, skill practice, and habitual self-monitoring [44]. These techniques were combined with nutrition education focused on maintaining a healthy diet and keeping healthy foods in the home. The curriculum had a module specifically dedicated to discussing improvements in the HFE which reviewed home food availability and the selection of healthier options at the store. Moreover, the intervention emphasized HFE improvements as part of the behavioral strategies promoted throughout multiple sessions. The curriculum content and corresponding activities were consistent at both study sites. Table 1 includes a list of the intervention topics and a brief description of their content. The main diet improvement targets were increasing the availability and intake of fruit and vegetables and reducing the availability and intake of sugar-containing foods and beverages. No interventions took place in the homes of participants during HFE data collection.

### 2.3. Measures

Data were collected in the households of participating families at baseline (after screening and consenting but before the start of intervention sessions) and within four weeks after completion of the intervention. Parent participants completed a survey to gather information on gender, age, income, number of people living in the household, employment status, education level, ethnicity, and if a household received any public food assistance (i.e., Women, Infants & Children [WIC], Supplemental Nutrition Assistance Program [SNAP]). HFE was assessed by trained research assistants using a modified version of the Home Food Inventory [29]. This tool captures the presence or absence of specific foods found in the main storage areas (i.e., home food availability), and whether foods are visible and readily obtainable on kitchen surfaces or without moving other items around in the refrigerator (i.e., kitchen and refrigerator accessibility, respectively). The inventory was modified by only including food categories within the main study outcomes of interest (fruits, vegetables, sugar-laden foods and beverages, and sugar-free beverages), and was culturally adapted to include foods commonly consumed by Hispanic individuals (e.g., pan dulce, aguas frescas). A section devoted to inventorying dry breakfast cereal was added. Dry cereal was categorized based on 2014 Arizona WIC guidelines as WIC-approved (low-sugar; no more than 6 grams of sugar per 1-ounce serving) or as sugar-laden (more than 6 grams of sugar per 1-ounce serving), regardless of fiber content [45]. Table 2 displays a list of the food items included in the modified inventory and used for the present analysis. The full instrument used for this study is available in Supplementary Material 2.

### 2.4. Analysis

Statistical analyses were conducted using IBM SPSS Statistics, version 21.0 (SPSS Inc., Chicago, IL, USA). All continuous variables are presented in text and tables as mean ± standard deviation (Mean ± SD) or frequency where appropriate. Data were summarized using descriptive statistics to capture the baseline characteristics of participants. Food availability values reported indicate the number (count) of different items present in a household at baseline or follow-up, and the difference between the two time points. Kitchen or refrigerator accessibility data represent the number of households that had each item being accessible (i.e., readily obtainable without moving other food items) at baseline or follow-up. Changes in home food availability were compared using a paired samples *t*-test for normally distributed variables (fruits and vegetables) or a Wilcoxon Signed Rank test for all other variables which were not normally distributed and/or could not be transformed (sugar-sweetened beverages, prepared desserts, candy, sugar-laden cereal, and high fiber/low sugar cereal). Vegetables were analyzed separately to include and exclude potatoes. Sugar-containing beverages, diet-beverages, and water were counted separately. Changes in kitchen and refrigerator accessibility are reported as the number of households that had those items readily obtainable, and were compared using a McNemar Test using the Quick Calcs calculator [46]. 

## 3. Results

### 3.1. Participant Sociodemographic Characteristics and Program Attendance

The mean age was 36.0 ± 5.5 years with an average household size of 2.4 ± 1.0 adults and 2.7 ± 1.1 children. The majority of participants were female (*n* = 21; 91%) and identified themselves as Hispanic (*n* = 18; 78%). Other participants self-identified as African-American (*n* = 1), Native American (*n* = 1), Caucasian (*n* = 2) and Somali (*n* = 1). Overall, the level of education was high school or lower for 52% of participants; 22% of participants were college graduates. Fewer than half of the participants reported working either full-time (22%) or part-time (17%); 39% of participants reported being homemakers. About 61% of households had an income less than USD 2000/month, and 39% of households reported to be receiving public food assistance. Participants’ mean attendance was 7.7 ± 1.2 sessions.

### 3.2. Changes in Food Availability

Changes in the number of available food items per household from baseline to follow-up are depicted in Table 3. There was a significant increase in the availability of fruit (1.7 items; *p* = 0.004) but not vegetables (*p* = 0.111), and significant reductions in sugar-sweetened beverages (−1.5 items; *p* = 0.004), available prepared desserts (−1.3 items; *p* = 0.005) and available types of candy (−1.4 items; *p* < 0.001). There were no significant differences in sugar-laden dry cereal availability (*p* = 0.090), but low-sugar cereal significantly increased (0.4 items; *p* = 0.033).

### 3.3. Changes in Kitchen and Refrigerator Food Accessibility

There were no changes in the number of households with readily accessible fresh fruit or vegetables, dry cereal, regular soda, candy, or prepared desserts within the kitchen (Table 4). At baseline, chocolate and strawberry flavored milk were accessible in seven and three households, respectively; those products were not accessible at follow-up (*p* = 0.023 for chocolate flavored milk; n.s. for strawberry flavored milk). Changes in refrigerator accessibility of beverages included significant increases in 100% fruit juice (+14 households; *p* = 0.001) and bottled/contained water (+13 households; *p* < 0.001), a non-significant reduction in fruit drinks and sports drinks (−7 households; *p* = 0.065), and no changes in regular or diet soda (*p* = 0.453 and *p* = 0.480, respectively). There were significant increases in refrigerator-accessible ready-to-eat fruits (+9 households; *p* = 0.022) and vegetables (+11 households; *p* = 0.007).

## 4. Discussion

Given that the HFE is a key influence on food choice and diet quality [30,31,32], effective intervention strategies to increase the intake of fruits and vegetables among children include those that target potential environmental determinants of intake, such as the availability and accessibility of these items in the household [47]. This is particularly important among Hispanic families, given their lower compliance with dietary guidance [16]. The current pilot study findings pose the possibility for community-based programs to improve diet quality by increasing home availability of fruits and WIC-approved cereal, while decreasing sugar-containing products. The intervention used for the current study emphasized HFE improvements as part of the behavioral strategies promoted throughout the program, particularly focusing on encouraging fruit and vegetable intake and reduced consumption of sugar-containing foods and beverages. Furthermore, the intervention had a session dedicated to discussing HFE improvements, including specific strategies to increase the home availability of fruit and vegetables, and the replacement of sugar-containing foods with more healthy options.

In this study, intervention participation resulted in increased home availability of fruit but not vegetables. Other interventions relying on self-reported data have also documented increased home availability of fruit or vegetables in households with children [26,35,48], with greater changes in fruit availability than vegetable availability [35]. Parents may be more likely to increase availability of fruit, as those tend to be more acceptable among children than vegetables, and do not require additional preparation (e.g., cooking) for consumption. Home environmental triggers may not impact fruit and vegetable consumption in comparable ways. Evidence suggests that placing a fruit bowl on the table may prompt increased fruit intake, while other factors such as culture-specific eating patterns practiced in the home may determine the amount of vegetables consumed at meal times [27]. This represents an opportunity to integrate more activities that focus on food preparation and cooking skills aimed at including more vegetables in meals in future interventions.

Although the current study did not compare dietary intake data to the observed changes in the HFE, previous research suggests that home food availability and accessibility play a role in consumption behaviors, food preferences, dietary quality, and weight status [30,32,48,49]. Homes with more fruits and vegetables available have been described as being overall more motivating and supportive for both adult and child fruit and vegetable intake when compared to homes with low fruit and vegetable availability [26,50]. This suggests the importance of combining parental encouragement for children to eat fruits and vegetables while also providing a home environment to encourage this behavior.

The current intervention resulted in lower home availability of sugar-sweetened beverages, prepared desserts, and candy. Home availability of sugar-containing foods and beverages has been reported to impact sugar intake and health outcomes [48,51]. However, a systematic review of studies assessing correlates or determinants of sugar-sweetened beverage consumption concluded that the evidence associating intake with home availability is equivocal [52]. Nevertheless, some interventions resulting in a lower home availability of sugar-containing foods have also reported reduced sugar intake [53]. Given the disparities in diet quality observed among underrepresented populations [54], strategies that reduce the availability of sugar-laden foods in the household may be good approaches to reduce overall sugar intake and improve diet quality. Replication studies are needed in order to compare results by differences in household racial and ethnic make-up. 

Whereas this study is novel and adds to the literature on the HFE, several potential strengths and limitations deserve mention. A major strength of the current study is that it assessed the HFE through open inventories conducted by research staff during home visits instead of self-reported data, to avoid recall and social-desirability biases [40]. However, social desirability bias could not be completely eliminated given that home visits were scheduled, and study participants could have altered or hidden food items and beverages in the home on observation day. Open inventories have not been used to assess the efficacy of a behavior change intervention. Though this study attempted to evaluate a potential causal relationship between the intervention and the HFE through the availability and accessibility of fruits, vegetables, and sugar-sweetened products, the quasi-experimental design of this pilot study prohibits us from drawing causal conclusions. Household characteristics (e.g., household size, physical layout of dining and kitchen areas, cultural practices) may also contribute to availability and consumption behaviors. However, the study was strengthened by precautions to limit additional influence on the HFE of participants, particularly through our efforts to reduce or eliminate social desirability bias by collecting data through open inventories. Research assistants were extensively trained in the use of the Home Food Inventory, but HFE data were not collected in duplicate due to budgetary constraints. The lack of inter-rater reliability data is a methodological limitation of the study. Only study participants who consented to the home visit were included in this analysis, therefore the study sample may not be representative of the general population. Moreover, although the original intent was to exclusively enroll Hispanic families in the study, participation of parents from other ethnic groups limits the generalizability of findings to the overall Hispanic population. Parent participant enrollment was based on their willingness and availability to attend sessions, rather than their involvement in food procurement for the household. Focusing on the caregiver with the primary responsibility on food purchasing and preparation may lead to a greater impact of future interventions. The current analysis did not control for additional factors that may have affected findings, including seasonality, holidays, household socioeconomic status, dietary restrictions from other family members not enrolled in the study, and parental education. Though several culturally-relevant items were added to the home food inventory prior to the start of the intervention, other popular items were not included (e.g., papaya, jicama, radish). The analysis was limited to assessing food availability and accessibility and did not confirm whether observed changes actually resulted in modified food intake. Feasibly data regarding intervention implementation were not collected. Future work should collect feasibility data in order to inform large scale implementation efforts. Finally, the study tool used to assess home food availability was not quantitative (i.e., it captures variety but not amount of food items maintained at home); this is a limitation for many validated inventory tools, including a tool comparably designed to measure household food availability among low-income Mexican families [55].

## 5. Conclusions

Results suggest that the current intervention resulted in favorable changes in the HFE among a sample of primarily Hispanic participants. Existing health education programs could benefit from focusing on modifying the HFE to promote healthier eating behaviors of families, and health behavior change interventions focused on modifying the HFE could utilize home visits as an additional feasible measure of program efficacy. In addition to focusing on how to improve the HFE, programs should also include strategies for navigating the food environment outside of the home. Future research should explore the relationship between HFE and intake, dietary quality, and weight, incorporate more food preparation activities aimed at including vegetables in family meals, and add strategies for sustaining intervention effects after the intervention is completed.

## Figures and Tables

**Table 1:** Outline of Intervention Topics.

Module	Topic(s)	Content
1	Introduction/Chronic disease risk reduction	Description of the program. Discussion about common chronic diseases related to lifestyle.
2	Overview of a healthy diet/Meal planning	Introduction of basic nutrition concepts related to dietary guidance. Recommendations for the preparation of a weekly meal plan and grocery list as a way to incorporate dietary guidance in the family meals and increase fruit/vegetable availability in the home.
3	Fats and sugars/Reading food labels	Discussion about the role of dietary fat and sugar in chronic disease risk. Review of the key items on the nutrition facts panel and examples of how to use them for making food choices.
4	Recipe modification/Portion control	Discussion of ways to modify recipes to reduce sugar and fat content and increase fruit/vegetable consumption. Review of standard portion sizes for commonly consumed foods.
5	Benefits of physical activity/Healthy foods on a budget	Discussion of the importance of physical activity as a way to reduce chronic disease risk. Discussion of strategies to purchase less expensive foods without compromising dietary quality.
6	Healthy home food environment/Smart shopping	Discussion of the importance of the home environment for making smart diet choices and for providing healthy foods for the family, and the benefits of having healthy options as the more easily accessible foods for children. Strategies for improving the home food environment by selecting healthier food options when shopping.
7	Involving the family/Family mealtime	Discussion of the importance of involving children and other family members in healthy food selection and preparation. Discussion of the importance of having meals as a family.
8	Why we eat: hunger vs. nourishment	Discussion of the difference between high-calorie and high-nutrient foods.
9	Physical activity and sedentary behaviors	Discussion of the role of physical activity and sedentary behaviors on health.
10	Final review and graduation	Question and answer session. Potluck to celebrate the end of the program.

**Table 2:** Food items included in the modified Home Food Inventory.

Food Category	Items Included
*Fruits and Vegetables*	
Fruits	Apples, apple sauce, apricots, avocado, bananas, blueberries, cranberries, dates, grapes, grapefruit, kiwi, lemons or limes, mango, melon, mixed fruit/fruit cocktail, nectarines, oranges, pears, peaches, pineapple, plums, prunes, raisins, raspberries, strawberries, tangerines/clementines
Vegetables	Asparagus, beets, bell peppers, broccoli, cabbage, cauliflower, carrots, celery, corn, cucumbers, green beans, lettuce, mushrooms, peas, potatoes, spinach/other greens, squash
*Sugar-containing products*	
Beverages	Soda, prepared iced teas and lemonades, sports drinks, fruit drinks, flavored milks, aguas frescas, energy drinks, 100% fruit juice
Prepared desserts	Cookies, cakes/cupcakes, muffins, brownies, other snack cakes, pastries/sweet rolls/donuts, flan, pan dulce, ice cream, pudding/jello
Candy	Chocolate, hard candy, gummy candy varieties, fruit rollups/fruit snacks or other fruit-based candy, chewy candy
Dry breakfast cereal	WIC-approved low sugar, sugar-laden breakfast cereal
*Sugar-free alternatives*	
Beverages	Diet soda, diet iced tea, water

**Table 3:** Changes in home availability of food items from baseline to post-intervention ^1^.

Food Group	Baseline	Follow-up	Difference	*p* Value
	items			
Fruit	7.7 (3.2)	9.4 (3.1)	1.7	**0.004**
Vegetables (excluding potatoes)	8.7 (2.9)	9.5 (2.8)	0.8	0.111
Sugar-sweetened beverages	3.2 (1.9)	1.7 (1.3)	−1.5	**0.004**
Prepared desserts	3.0 (2.0)	1.7 (1.3)	−1.3	**0.005**
Candy	2.0 (1.7)	0.6 (0.7)	−1.4	**<0.001**
Sugar-laden cereal	2.4 (2.1)	1.8 (1.5)	−0.6	0.090
High fiber/low sugar cereal	2.3 (1.4)	2.7 (1.4)	0.4	**0.033**

^1^ Values are presented as Mean (SD) and represent the number of items within each category present in the household (*n* = 23). Mean values were compared using a paired samples *t*-test for normally distributed variables (fruit and vegetables), or a Wilcoxon Signed rank test for all other variables. Emboldened *p* values indicate statistical significance.

**Table 4:** Changes in kitchen and refrigerator accessibility from baseline to follow-up ^1^.

Food Item	Pre	Post	Difference	*p* Value
			households	
Kitchen accessibility				
Fresh fruit	19	22	3	0.250
Fresh vegetables	5	6	1	1.000
Dry cereal	11	8	−3	0.375
Regular soda pop	3	2	−1	1.000
Candy	5	3	−2	0.688
Regular prepared desserts ^2^	3	7	4	0.289
Refrigerator accessibility				
Flavored milk (chocolate)	7	0	−7	0.023
Flavored milk (strawberry)	3	0	−3	0.248
100% fruit juice	3	17	14	**0.001**
Fruit drinks/sports drinks	13	6	−7	0.065
Regular soda pop	9	6	−3	0.453
Diet soda pop	2	0	−2	0.480
Bottled/contained water	9	22	13	**<0.001**
Fresh ready-to-eat vegetables	8	19	11	**0.007**
Fresh ready-to-eat fruit	8	17	9	**0.022**

^1^ Values represent the frequency of households in which each item was accessible in the kitchen or refrigerator (*n* = 23). The frequency of households having an item accessible in the kitchen or refrigerator was compared using a McNemar Test. Emboldened *p* values indicate statistical significance; ^2^ Accessible regular prepared desserts included cookies, cakes, cupcakes, and muffins. Emboldened *p* values indicate statistical significance.

## Supplementary Materials

The following are available online at https://www.mdpi.com/1660-4601/17/22/8327/s1.
