# Supplementary material for: Effects of a Community-Based Pilot Intervention on Home Food Availability among U.S. Households
Source: Int J Environ Res Public Health. 2020 Nov 11;17(22):8327. doi: 10.3390/ijerph17228327 (PMC7697825; doi:10.3390/ijerph17228327)
Supplement: Supplementary file 1 [file ijerph-17-08327-s001.zip › ijerph-971054-Supplement1-send proof.pdf]

Supplement 1. Eligibility Verification Questions

Do you have a child that that is 6-11 years old?

☐ YES ☐ NO, STOP

Are you able to participate in English/Spanish language classes that will be going over a variety of topics related to health, nutrition, and physical activity?

☐ YES ☐ NO, STOP

Are you available to participate in this study each week at ________________ (time) on __________________ (day)?

☐ YES ☐ NO, STOP

Do you have a medical condition that requires you to follow a specific diet?

☐ YES ☐ NO, STOP

Are you currently participating in a diet modification program?

☐ YES ☐ NO, STOP

Are you currently pregnant?

☐ YES ☐ NO, STOP

Do you consume more than 5 servings of fruits and vegetables daily?

☐ YES ☐ NO, STOP

**Is patient eligible for participation?**	**YES** ☐	**NO** ☐ **(STOP)**
