# Supplementary material for: Effects of a Community-Based Pilot Intervention on Home Food Availability among U.S. Households
Source: Int J Environ Res Public Health. 2020 Nov 11;17(22):8327. doi: 10.3390/ijerph17228327 (PMC7697825; doi:10.3390/ijerph17228327)
Supplement: Supplementary file 1 [file ijerph-17-08327-s001.zip › ijerph-971054-Supplement2-send proof.pdf]

Supplement 2. Modified Home Food Inventory

Home Food Inventory (AFL Modified)

|___| Confirmed if participant has an additional fridge/freezer outside of kitchen

START TIME: ____________AM/PM

1. <u>RESEARCH ASSISTANTS:</u> Please look around the kitchen (countertop, top of refrigerator, table) and indicate which of the following items are visible and readily accessible.

Yes	No	
1	0	a. Fresh fruit
1	0	b. Dried Fruit
1	0	c. Canned fruit
1	0	d. Fresh vegetables
1	0	e. Canned vegetables
1	0	f. Regular snack crackers, pretzels, chips, popcorn
1	0	g. Reduced-fat snack crackers, pretzels, chips, popcorn
1	0	h. Dry cereal
1	0	i. Bread or rolls
1	0	j. Regular soda pop
1	0	k. Diet soda pop
1	0	l. Candy
1	0	m. Regular cookies, cake, cupcakes, muffins
1	0	n. Reduced-fat cookies, cake, cupcakes, muffins

2. Now please open the refrigerator (kitchen). Which of the following items can you see without moving items around?

Yes	No	
1	0	a. Plain whole milk
1	0	b. Plain 2% milk
1	0	c. Plain 1% milk
1	0	d. Plain Skim milk
1	0	e. Flavored milk (chocolate)
1	0	f. Favored milk (strawberry)
1	0	g. Flavored milk (other flavors)
1	0	h. Non-dairy milks (soy, rice, almond, etc)
1	0	i. 100% fruit juice (any flavor)
1	0	j. Diet 100% fruit juice (any flavor)
1	0	k. Fruit drinks/sports drinks (not 100% juice)
1	0	l. Diet fruit drinks/sports drinks (not 100% juice)
1	0	m. Regular soda pop
1	0	n Diet soda pop
1	0	o. Bottled/contained water
1	0	p. Reduced-fat yogurt drinks
1	0	q. Fresh ready-to-eat vegetables
1	0	r. Fresh ready-to-eat fruit

<u>RESEARCH ASSISTANTS:</u> Now look in all areas in of the home where the household stores food, including the refrigerator, freezer, pantries, cupboards, and other storage areas (list follows in that order). Please check "yes" or "no" to each of the food product/item/category below. Check "yes" to a food product/item/category if it is present anywhere in the home (opened or unopened) as you are completing this form. Check "no" to a food product/item/category if it is not present anywhere in the home when completing this form.

Note, please mark whether each vegetable present is fresh, canned or frozen ***(mark all that apply).*** *For example, if the household has both fresh and canned asparagus, you would check "yes" to asparagus and check in both the fresh and canned columns.*

3. <u>Vegetables</u>

Yes	No		Fresh	Can/Jar	Frozen
			(Mark all that apply)		
1	0	a. Asparagus	1	1	1
1	0	b. Beets	1	1	1
1	0	c. Bell peppers (example: green, red)	1	1	1
1	0	d. Broccoli	1	1	1
1	0	e. Cabbage	1	1	1
1	0	f. Cauliflower	1	1	1
1	0	g. Carrots	1	1	1
1	0	h. Celery	1	1	1
1	0	i. Corn	1	1	1
1	0	j. Cucumbers	1	1	1
1	0	k. Green beans	1	1	1
1	0	l. Lettuce (example: romaine, endive)	1	1	1
1	0	m. Mushrooms	1	1	1
1	0	n. Peas	1	1	1
1	0	o. Potatoes	1	1	1
1	0	p. Spinach/other greens (collard)	1	1	1
1	0	q. Squash (example: butternut, zucchini)	1	1	1
1	0	r. Sweet potatoes	1	1	1
1	0	s. Tomatoes	1	1	1

1	0	t. Mixed vegetables	1	1	1

Note, please check whether each fruit present is fresh, canned, frozen, or dried (*mark all that apply*). *For example, if the household has both fresh and frozen blueberries, you would check "yes" to blueberries and check in both the fresh and frozen columns.*

4. Fruit

Yes	No		Fresh	Can/Jar	Frozen	Dried
			(Mark all that apply)			
1	0	a. Apples	1	1	1	1
1	0	b. Apple sauce	1	1	1	1
1	0	c. Apricots	1	1	1	1
1	0	d. Avocado	1	1	1	1
1	0	e. Bananas	1	1	1	1
1	0	f. Blueberries	1	1	1	1
1	0	g. Cranberries	1	1	1	1
1	0	h. Dates	1	1	1	1
1	0	i. Grapes (red or green)	1	1	1	1
1	0	j. Grapefruit	1	1	1	1
1	0	k. Kiwi	1	1	1	1
1	0	l. Lemons or limes	1	1	1	1
1	0	m. Mango	1	1	1	1
1	0	n. Melons (example: watermelon)	1	1	1	1
1	0	o. Mixed fruit/fruit cocktail	1	1	1	1
1	0	p. Nectarines	1	1	1	1
1	0	q. Oranges	1	1	1	1

Go to next page.

Note, please check

whether each prepared dessert type present is homemade or store-bought (mark all that

1	0	r. Pears	1	1	1	1	**apply).** *For*
1	0	s. Peaches	1	1	1	1	
1	0	t. Pineapple	1	1	1	1	
1	0	u. Plums	1	1	1	1	
1	0	v. Prunes	1	1	1	1	
1	0	w. Raisins	1	1	1	1	
1	0	x. Raspberries	1	1	1	1	
1	0	y. Strawberries	1	1	1	1	
1	0	z. Tangerines/Clementines	1	1	1	1	

example, if the household has both homemade and store-bought chocolate chip cookies, you would check "yes" to regular cookies and check in both the store bought and homemade columns.

5. **Prepared Desserts** ***(do not count boxed mixes that are not prepared)***

Yes	No		Store-bought	Homemade
			(Mark all that apply)	
1	0	a. Regular cookies (any flavor/variety)	1	1
1	0	b. Reduced-fat cookies (any flavor/variety)	1	1
1	0	c. Regular cake/cupcakes (any flavor)	1	1
1	0	d. Reduced-fat cake/cupcakes (any flavor)	1	1
1	0	e. Regular muffins (any flavor/variety)	1	1
1	0	f. Brownies/bars (any variety)	1	1
1	0	g. Other snack cakes (any variety)	1	1
1	0	h. Pastry, sweet rolls, donuts	1	1
1	0	i. Flan	1	1
1	0	j. Pan dulce (sweet bread)	1	1

1	0	k. Ice cream (any variety or flavor)	1	1
1	0	l. Pudding or Jello (any variety or flavor)	1	1

6. **<u>Beverages</u>** ***<u>(do not include alcoholic beverages)</u>***

Yes	No	
1	0	a. Regular soda pop (any variety, flavor)
1	0	b. Diet soda pop (any variety, flavor)
1	0	c. Prepared iced teas or lemonade (example: Snapple)
1	0	d. Prepared light iced teas or lemonade (example: diet Snapple)
1	0	e. Sports drinks (example: Gatorade, Vitamin Water)
1	0	f. Diet sports drinks
1	0	g. 100% fruit juice (labeled as 100% juice)
1	0	h. Diet 100% fruit juice
1	0	i. Fruit drinks (example: <100% juice, Capri Sun)
1	0	j. Diet fruit drinks
1	0	k. Bottled water (unsweetened, any variety, flavor)
1	0	l. Plain whole milk
1	0	m. Plain 2% milk
1	0	n. Plain 1% milk
1	0	o. Plain Skim milk
1	0	p. Flavored milk (chocolate)
1	0	q. Favored milk (strawberry)
1	0	r. Flavored milk (other flavors)

1	0	s. Non-dairy milks (soy, rice, almond, etc)
1	0	t. Energy Drink (any variety or flavor)
1	0	u. Aguas frescas

7. **Candy**

Yes	No	
1	0	a. Chocolate candy (any variety, except chocolate exclusively for baking)
1	0	b. Hard candy
1	0	c. Gummis
1	0	d. Fruit rollups, fruit snacks or other fruit based candy
1	0	e. Chewy candy (example: Skittles, caramel, tamarindo)

8. **Dry Cereal**

Note, the goal in this section is to assess whether cereals follow WIC-Guidelines, which includes those that have no more than 6g of sugar per 1 dry oz. Please make note of all cereals in the household.

Does the household have available dry cereal with more than 6 grams of sugar per serving? **Yes No**

1	0

If yes, please list below. Examples: Frosted Flakes, Lucky Charms, Honey Smacks, Fruit Loops, Captain Crunch, etc.

**Name of cereal** (Include variety i.e. Honey Bunches of Oats w/ almonds)	**Brand name**	**Sugar (g/serving)**	**Serving size (g)**	**Box size**

Does the household have available dry cereal with 6 grams or less of sugar per serving?

**Yes**	**No**
1	0

If yes, please list below. Examples include plain Cheerios, Kix, Chex, Shredded Wheat, Plain Rice Krispies, etc.

**Name of cereal** (Include variety i.e. Honey Bunches of Oats w/ almonds)	**Brand name**	**Sugar (g/serving)**	**Serving size (g)**	**Box size**

9. When was your last food-shopping trip? ______________________________

a. How many days ago was that? ______________________________

10. Where did you shop on your last food-shopping trip?____________________

11. On your last grocery shopping trip, did you make any purchases that are not typical for your family that are still in the home? (e.g. birthday party supplies, groceries for a friend, etc.)

Yes No

If yes, please elaborate____

12. Is this the usual amount of food you would keep in your home?

Yes No

If no, please elaborate____

13. Where do you buy *most* of your food? ____________________________________

14. How often do you shop at other grocery stores, markets, etc? _________________

a. Would you call that daily, weekly, monthly, rarely, or never? ______________

15. How many individuals live in your household? ______________________________

16. How was your overall experience about participating in the home visit?

Strongly disliked
Disliked
Neutral
Liked
Strongly Liked

17. What was your family's attitude towards your participation in the home visit?

Strongly disliked
Disliked
Neutral
Liked
Strongly Liked

18. How likely would you be to allow a home visit in your household again?

Very Unlikely
Unlikely
Unsure
Likely
Very Likely

Please provide any additional comments or suggestions about this home visit.

____
____
____
____

This is the end of the food inventory.

Adapted from: Fulkerson JA, Nelson MC, Lytle LA, Moe S, Heitzler C, Pasch KE. The validation of a home food inventory. International Journal of Behavioral Nutrition and Physical Activity, 2008, 5;55.
